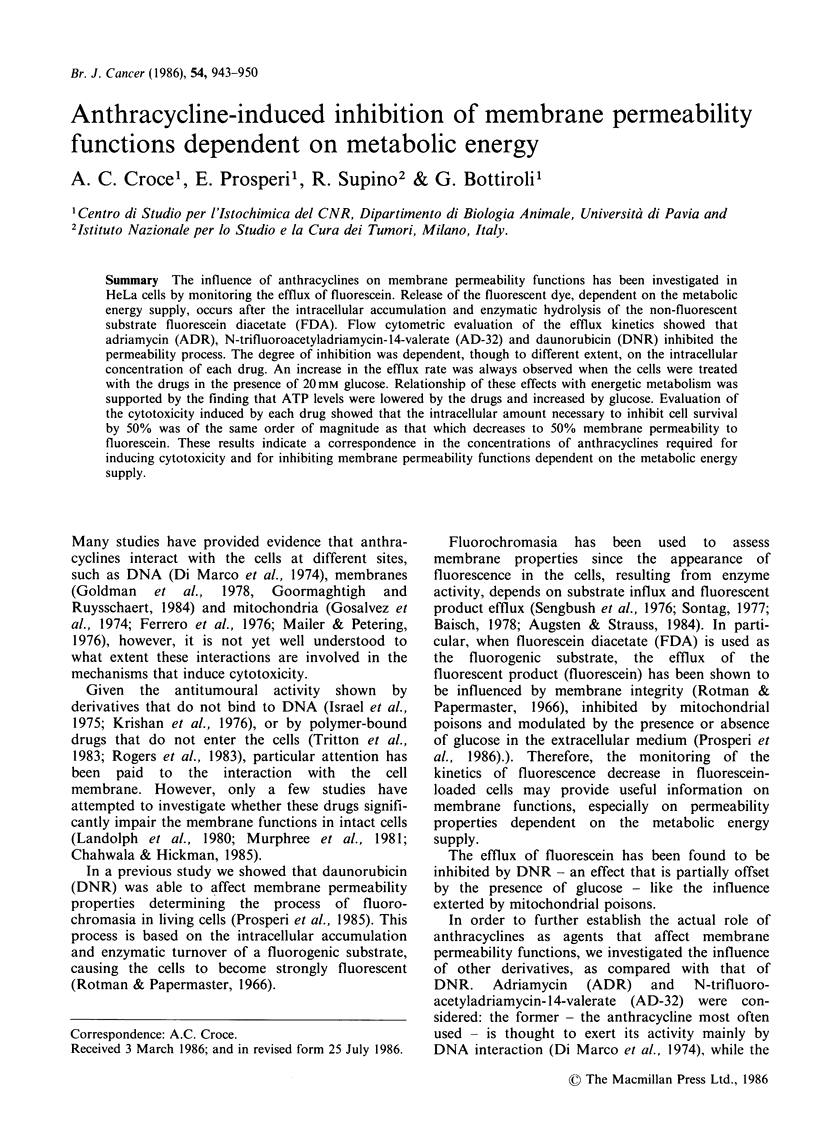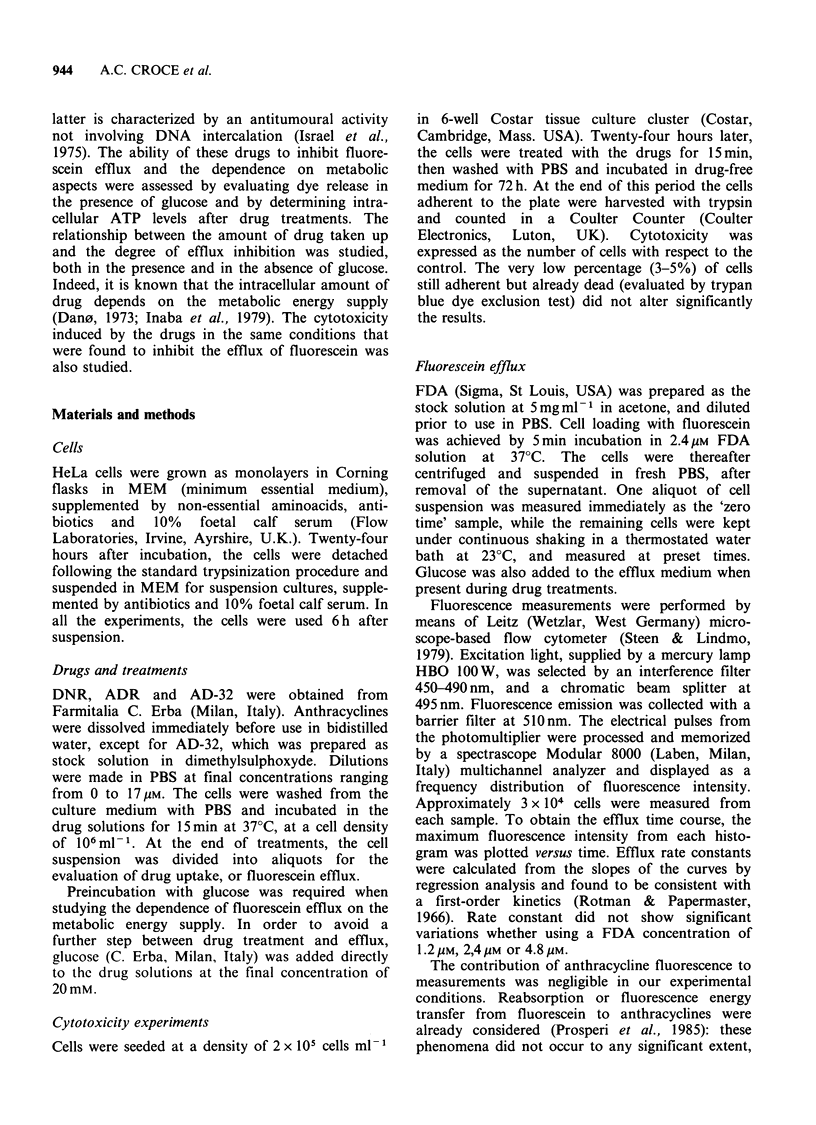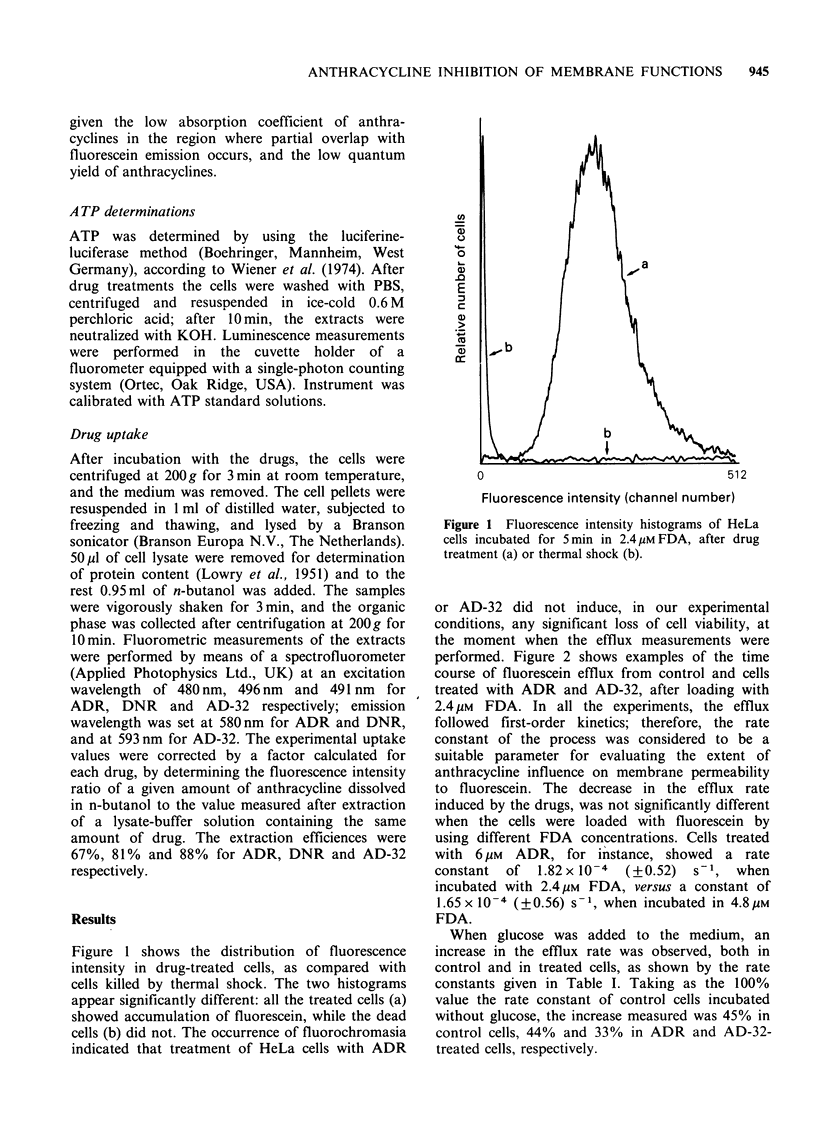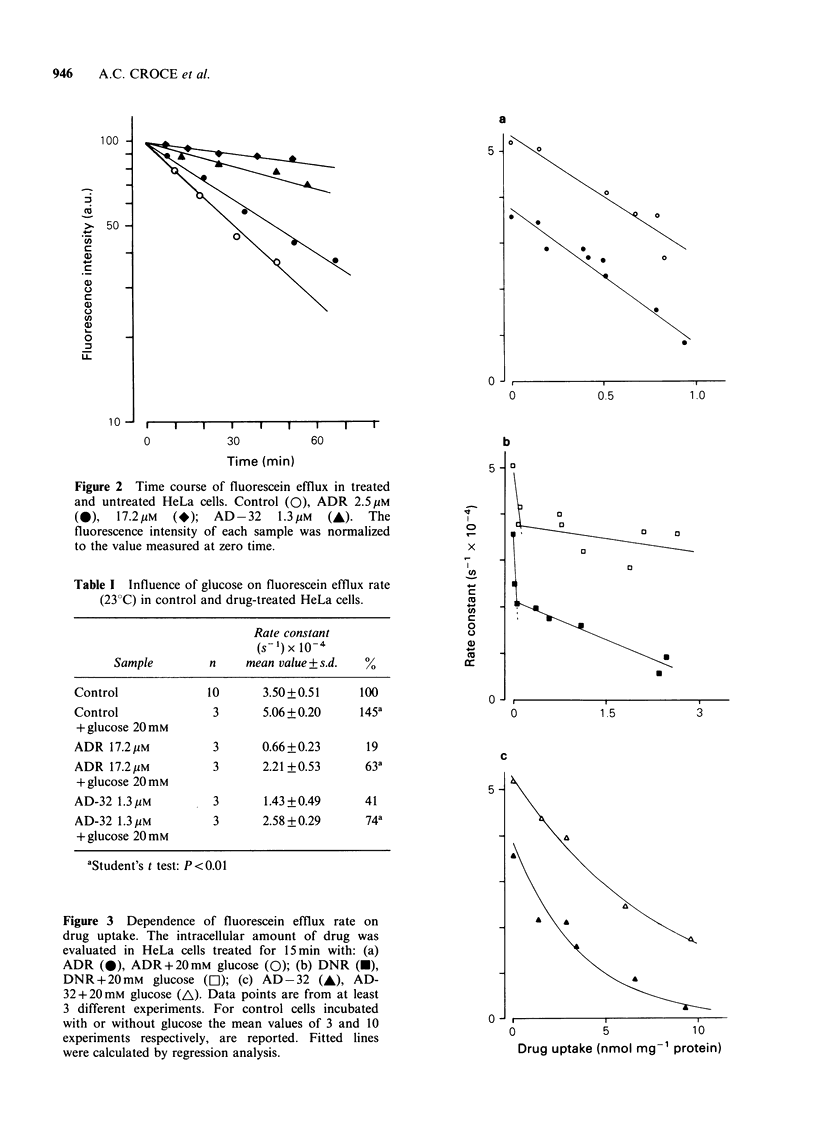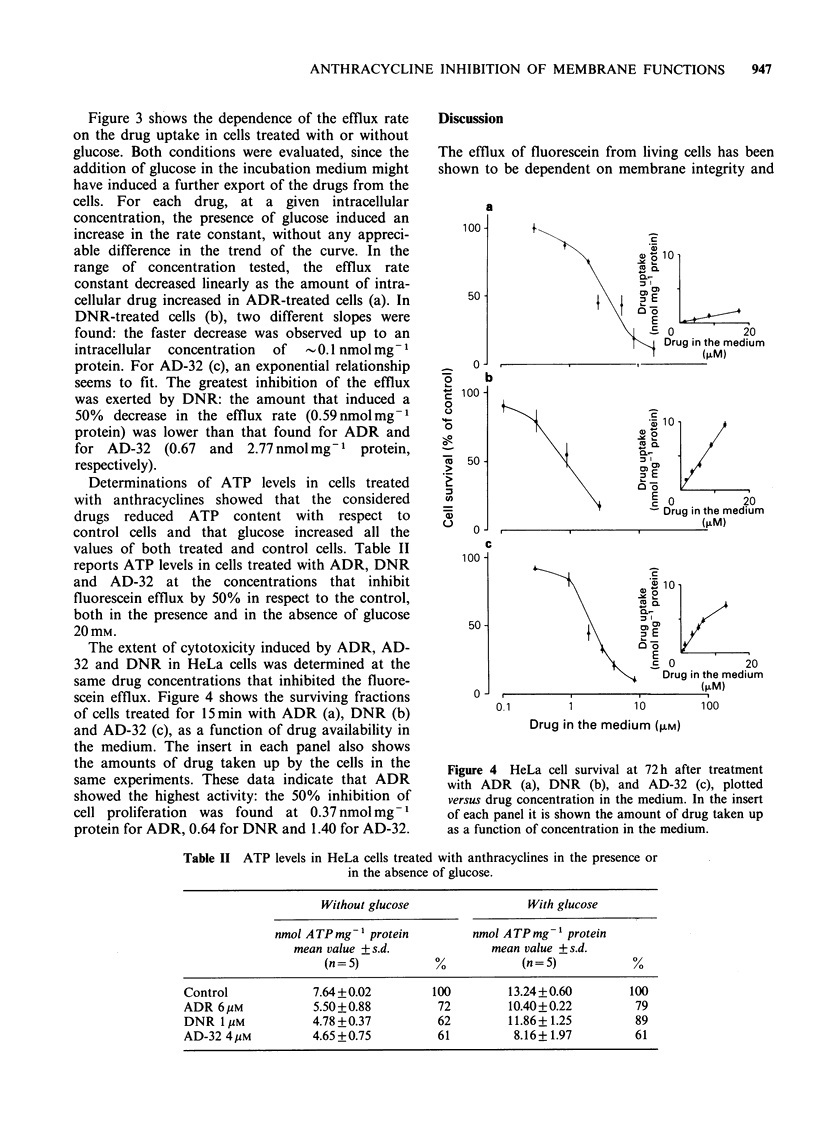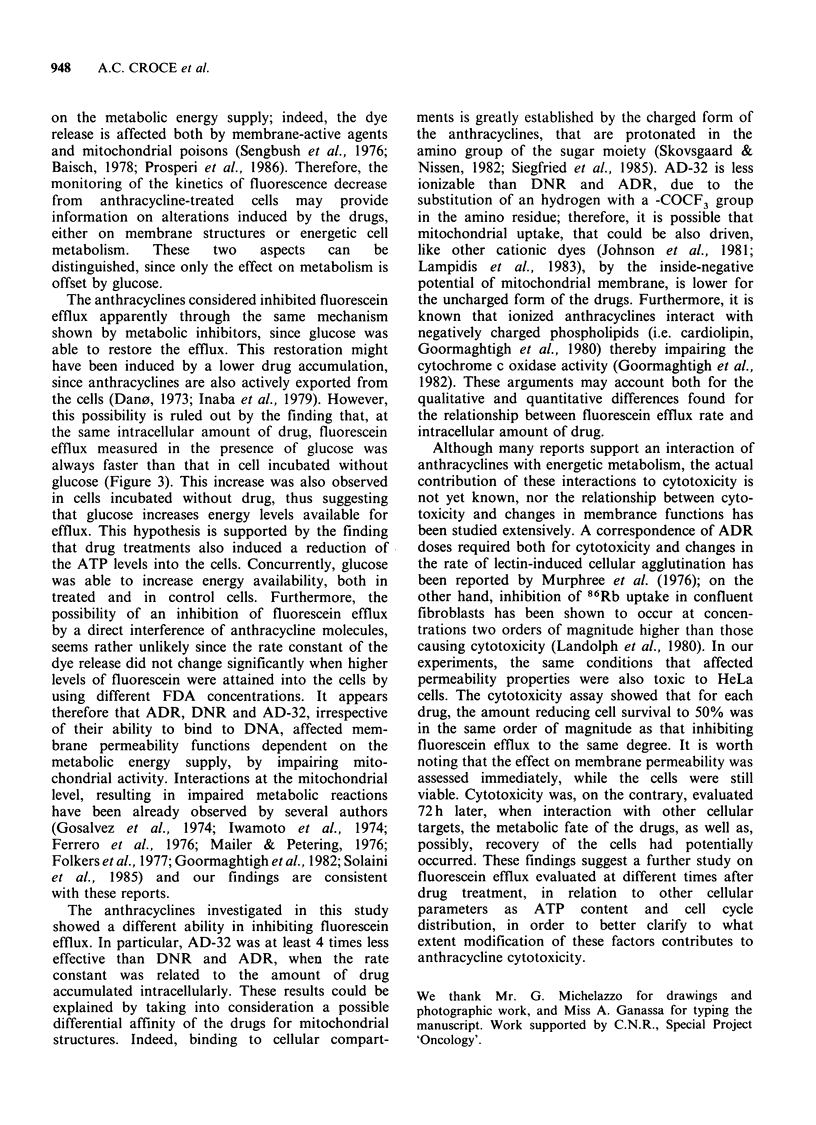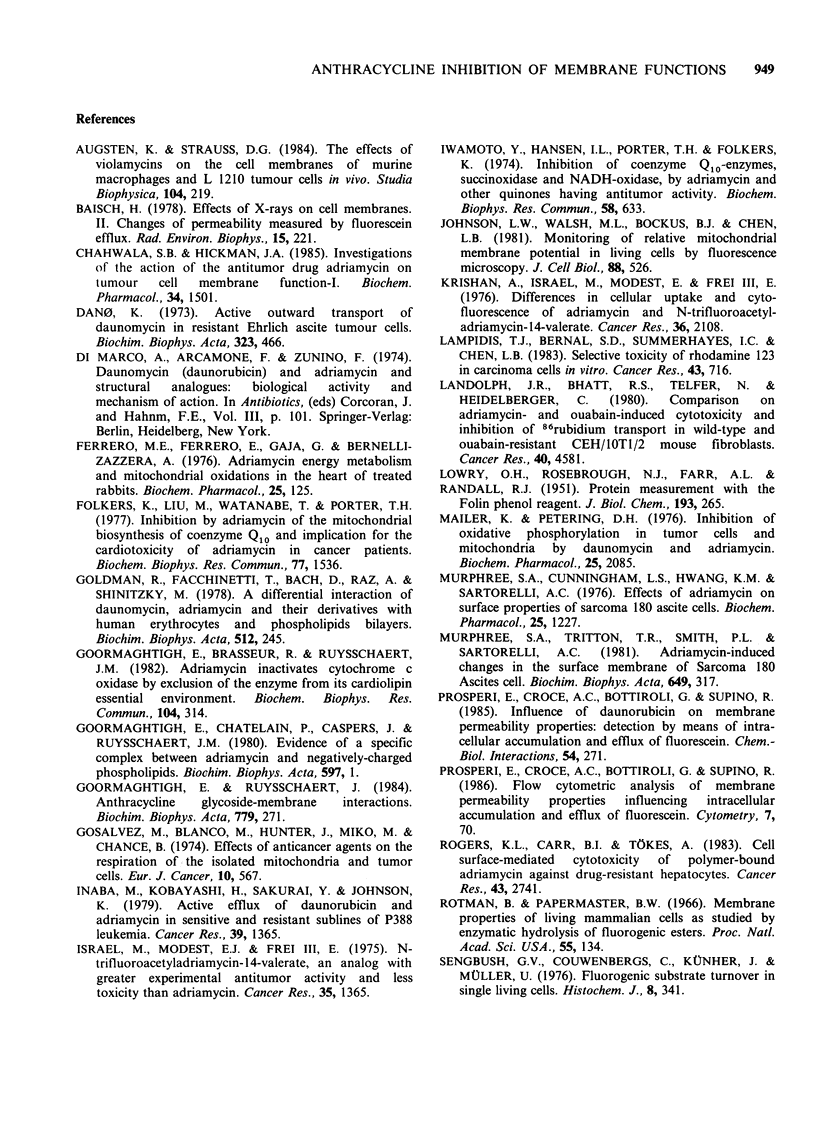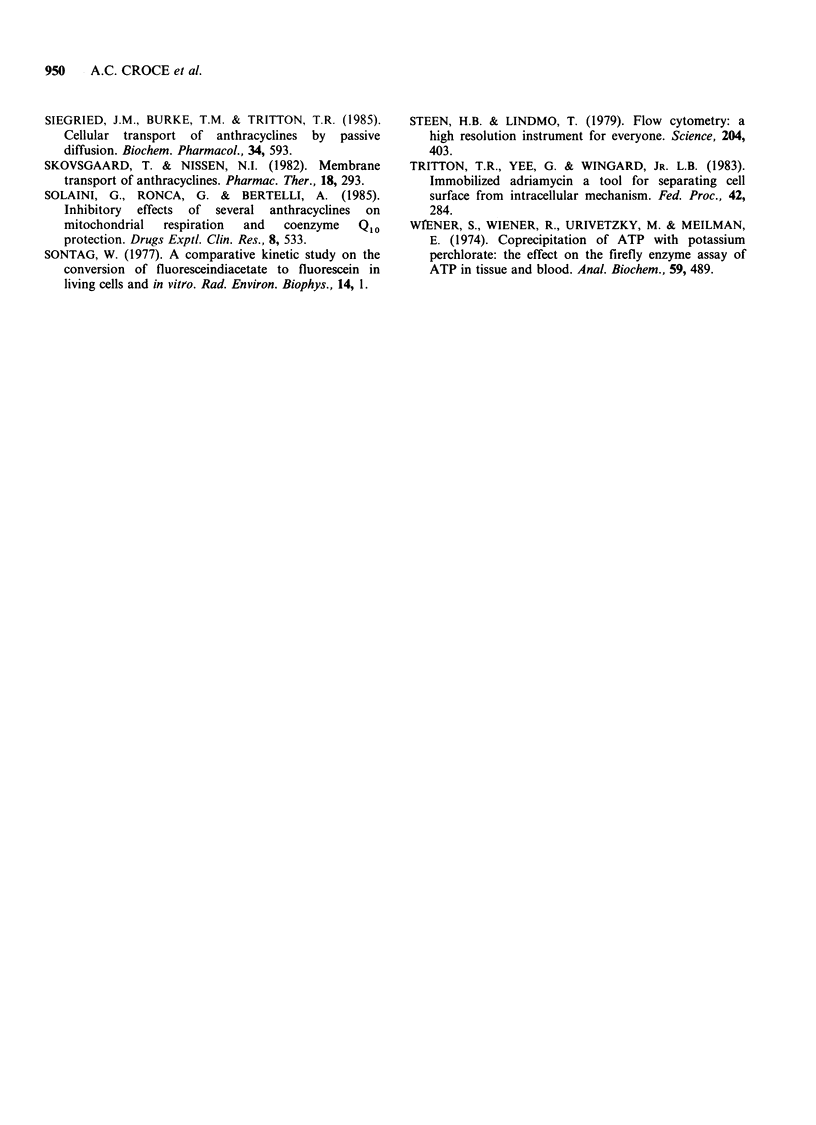# Anthracycline-induced inhibition of membrane permeability functions dependent on metabolic energy.

**DOI:** 10.1038/bjc.1986.265

**Published:** 1986-12

**Authors:** A. C. Croce, E. Prosperi, R. Supino, G. Bottiroli

## Abstract

The influence of anthracyclines on membrane permeability functions has been investigated in HeLa cells by monitoring the efflux of fluorescein. Release of the fluorescent dye, dependent on the metabolic energy supply, occurs after the intracellular accumulation and enzymatic hydrolysis of the non-fluorescent substrate fluorescein diacetate (FDA). Flow cytometric evaluation of the efflux kinetics showed that adriamycin (ADR), N-trifluoroacetyladriamycin-14-valerate (AD-32) and daunorubicin (DNR) inhibited the permeability process. The degree of inhibition was dependent, though to different extent, on the intracellular concentration of each drug. An increase in the efflux rate was always observed when the cells were treated with the drugs in the presence of 20 mM glucose. Relationship of these effects with energetic metabolism was supported by the finding that ATP levels were lowered by the drugs and increased by glucose. Evaluation of the cytotoxicity induced by each drug showed that the intracellular amount necessary to inhibit cell survival by 50% was of the same order of magnitude as that which decreases to 50% membrane permeability to fluorescein. These results indicate a correspondence in the concentrations of anthracyclines required for inducing cytotoxicity and for inhibiting membrane permeability functions dependent on the metabolic energy supply.


Br. J. Cancer (1986), 54, 943–950

### Anthracycline-induced inhibition of membrane permeability functions dependent on metabolic energy

A. C. Croce[1], E. Prosperi[1], R. Supino[2] & G. Bottiroli[1]

[1]*Centro di Studio per l'Istochimica del CNR, Dipartimento di Biologia Animale, Università di Pavia and* [2]*Istituto Nazionale per lo Studio e la Cura dei Tumori, Milano, Italy.*

**Summary** The influence of anthracyclines on membrane permeability functions has been investigated in HeLa cells by monitoring the efflux of fluorescein. Release of the fluorescent dye, dependent on the metabolic energy supply, occurs after the intracellular accumulation and enzymatic hydrolysis of the non-fluorescent substrate fluorescein diacetate (FDA). Flow cytometric evaluation of the efflux kinetics showed that adriamycin (ADR), N-trifluoroacetyladriamycin-14-valerate (AD-32) and daunorubicin (DNR) inhibited the permeability process. The degree of inhibition was dependent, though to different extent, on the intracellular concentration of each drug. An increase in the efflux rate was always observed when the cells were treated with the drugs in the presence of 20 mM glucose. Relationship of these effects with energetic metabolism was supported by the finding that ATP levels were lowered by the drugs and increased by glucose. Evaluation of the cytotoxicity induced by each drug showed that the intracellular amount necessary to inhibit cell survival by 50% was of the same order of magnitude as that which decreases to 50% membrane permeability to fluorescein. These results indicate a correspondence in the concentrations of anthracyclines required for inducing cytotoxicity and for inhibiting membrane permeability functions dependent on the metabolic energy supply.

Many studies have provided evidence that anthracyclines interact with the cells at different sites, such as DNA (Di Marco *et al.*, 1974), membranes (Goldman *et al.*, 1978, Goormaghtigh and Ruysschaert, 1984) and mitochondria (Gosalvez *et al.*, 1974; Ferrero *et al.*, 1976; Mailer & Petering, 1976), however, it is not yet well understood to what extent these interactions are involved in the mechanisms that induce cytotoxicity.

Given the antitumoural activity shown by derivatives that do not bind to DNA (Israel *et al.*, 1975; Krishan *et al.*, 1976), or by polymer-bound drugs that do not enter the cells (Tritton *et al.*, 1983; Rogers *et al.*, 1983), particular attention has been paid to the interaction with the cell membrane. However, only a few studies have attempted to investigate whether these drugs significantly impair the membrane functions in intact cells (Landolph *et al.*, 1980; Murphree *et al.*, 1981; Chahwala & Hickman, 1985).

In a previous study we showed that daunorubicin (DNR) was able to affect membrane permeability properties determining the process of fluorochromasia in living cells (Prosperi *et al.*, 1985). This process is based on the intracellular accumulation and enzymatic turnover of a fluorogenic substrate, causing the cells to become strongly fluorescent (Rotman & Papermaster, 1966).

Correspondence: A.C. Croce.
Received 3 March 1986; and in revised form 25 July 1986.

Fluorochromasia has been used to assess membrane properties since the appearance of fluorescence in the cells, resulting from enzyme activity, depends on substrate influx and fluorescent product efflux (Sengbush *et al.*, 1976; Sontag, 1977; Baisch, 1978; Augsten & Strauss, 1984). In particular, when fluorescein diacetate (FDA) is used as the fluorogenic substrate, the efflux of the fluorescent product (fluorescein) has been shown to be influenced by membrane integrity (Rotman & Papermaster, 1966), inhibited by mitochondrial poisons and modulated by the presence or absence of glucose in the extracellular medium (Prosperi *et al.*, 1986).). Therefore, the monitoring of the kinetics of fluorescence decrease in fluorescein-loaded cells may provide useful information on membrane functions, especially on permeability properties dependent on the metabolic energy supply.

The efflux of fluorescein has been found to be inhibited by DNR – an effect that is partially offset by the presence of glucose – like the influence exterted by mitochondrial poisons.

In order to further establish the actual role of anthracyclines as agents that affect membrane permeability functions, we investigated the influence of other derivatives, as compared with that of DNR. Adriamycin (ADR) and N-trifluoroacetyladriamycin-14-valerate (AD-32) were considered: the former – the anthracycline most often used – is thought to exert its activity mainly by DNA interaction (Di Marco *et al.*, 1974), while the

© The Macmillan Press Ltd., 1986

latter is characterized by an antitumoural activity not involving DNA intercalation (Israel et al., 1975). The ability of these drugs to inhibit fluorescein efflux and the dependence on metabolic aspects were assessed by evaluating dye release in the presence of glucose and by determining intracellular ATP levels after drug treatments. The relationship between the amount of drug taken up and the degree of efflux inhibition was studied, both in the presence and in the absence of glucose. Indeed, it is known that the intracellular amount of drug depends on the metabolic energy supply (Danø, 1973; Inaba et al., 1979). The cytotoxicity induced by the drugs in the same conditions that were found to inhibit the efflux of fluorescein was also studied.

### Materials and methods

### Cells

HeLa cells were grown as monolayers in Corning flasks in MEM (minimum essential medium), supplemented by non-essential aminoacids, antibiotics and 10% foetal calf serum (Flow Laboratories, Irvine, Ayrshire, U.K.). Twenty-four hours after incubation, the cells were detached following the standard trypsinization procedure and suspended in MEM for suspension cultures, supplemented by antibiotics and 10% foetal calf serum. In all the experiments, the cells were used 6 h after suspension.

### Drugs and treatments

DNR, ADR and AD-32 were obtained from Farmitalia C. Erba (Milan, Italy). Anthracyclines were dissolved immediately before use in bidistilled water, except for AD-32, which was prepared as stock solution in dimethylsulphoxyde. Dilutions were made in PBS at final concentrations ranging from 0 to 17 $\mu$M. The cells were washed from the culture medium with PBS and incubated in the drug solutions for 15 min at 37°C, at a cell density of $10^6$ ml$^{-1}$. At the end of treatments, the cell suspension was divided into aliquots for the evaluation of drug uptake, or fluorescein efflux.

Preincubation with glucose was required when studying the dependence of fluorescein efflux on the metabolic energy supply. In order to avoid a further step between drug treatment and efflux, glucose (C. Erba, Milan, Italy) was added directly to the drug solutions at the final concentration of 20 mM.

### Cytotoxicity experiments

Cells were seeded at a density of $2 \times 10^5$ cells ml$^{-1}$ in 6-well Costar tissue culture cluster (Costar, Cambridge, Mass. USA). Twenty-four hours later, the cells were treated with the drugs for 15 min, then washed with PBS and incubated in drug-free medium for 72 h. At the end of this period the cells adherent to the plate were harvested with trypsin and counted in a Coulter Counter (Coulter Electronics, Luton, UK). Cytotoxicity was expressed as the number of cells with respect to the control. The very low percentage (3–5%) of cells still adherent but already dead (evaluated by trypan blue dye exclusion test) did not alter significantly the results.

### Fluorescein efflux

FDA (Sigma, St Louis, USA) was prepared as the stock solution at 5 mg ml$^{-1}$ in acetone, and diluted prior to use in PBS. Cell loading with fluorescein was achieved by 5 min incubation in 2.4 $\mu$M FDA solution at 37°C. The cells were thereafter centrifuged and suspended in fresh PBS, after removal of the supernatant. One aliquot of cell suspension was measured immediately as the 'zero time' sample, while the remaining cells were kept under continuous shaking in a thermostated water bath at 23°C, and measured at preset times. Glucose was also added to the efflux medium when present during drug treatments.

Fluorescence measurements were performed by means of Leitz (Wetzlar, West Germany) microscope-based flow cytometer (Steen & Lindmo, 1979). Excitation light, supplied by a mercury lamp HBO 100 W, was selected by an interference filter 450–490 nm, and a chromatic beam splitter at 495 nm. Fluorescence emission was collected with a barrier filter at 510 nm. The electrical pulses from the photomultiplier were processed and memorized by a spectrascope Modular 8000 (Laben, Milan, Italy) multichannel analyzer and displayed as a frequency distribution of fluorescence intensity. Approximately $3 \times 10^4$ cells were measured from each sample. To obtain the efflux time course, the maximum fluorescence intensity from each histogram was plotted *versus* time. Efflux rate constants were calculated from the slopes of the curves by regression analysis and found to be consistent with a first-order kinetics (Rotman & Papermaster, 1966). Rate constant did not show significant variations whether using a FDA concentration of 1.2 $\mu$M, 2,4 $\mu$M or 4.8 $\mu$M.

The contribution of anthracycline fluorescence to measurements was negligible in our experimental conditions. Reabsorption or fluorescence energy transfer from fluorescein to anthracyclines were already considered (Prosperi et al., 1985): these phenomena did not occur to any significant extent, given the low absorption coefficient of anthracyclines in the region where partial overlap with fluorescein emission occurs, and the low quantum yield of anthracyclines.

### *ATP determinations*

ATP was determined by using the luciferine-luciferase method (Boehringer, Mannheim, West Germany), according to Wiener *et al.* (1974). After drug treatments the cells were washed with PBS, centrifuged and resuspended in ice-cold 0.6 M perchloric acid; after 10 min, the extracts were neutralized with KOH. Luminescence measurements were performed in the cuvette holder of a fluorometer equipped with a single-photon counting system (Ortec, Oak Ridge, USA). Instrument was calibrated with ATP standard solutions.

### *Drug uptake*

After incubation with the drugs, the cells were centrifuged at 200 *g* for 3 min at room temperature, and the medium was removed. The cell pellets were resuspended in 1 ml of distilled water, subjected to freezing and thawing, and lysed by a Branson sonicator (Branson Europa N.V., The Netherlands). 50 $\mu$l of cell lysate were removed for determination of protein content (Lowry *et al.*, 1951) and to the rest 0.95 ml of *n*-butanol was added. The samples were vigorously shaken for 3 min, and the organic phase was collected after centrifugation at 200 *g* for 10 min. Fluorometric measurements of the extracts were performed by means of a spectrofluorometer (Applied Photophysics Ltd., UK) at an excitation wavelength of 480 nm, 496 nm and 491 nm for ADR, DNR and AD-32 respectively; emission wavelength was set at 580 nm for ADR and DNR, and at 593 nm for AD-32. The experimental uptake values were corrected by a factor calculated for each drug, by determining the fluorescence intensity ratio of a given amount of anthracycline dissolved in n-butanol to the value measured after extraction of a lysate-buffer solution containing the same amount of drug. The extraction efficiences were 67%, 81% and 88% for ADR, DNR and AD-32 respectively.

### Results

Figure 1 shows the distribution of fluorescence intensity in drug-treated cells, as compared with cells killed by thermal shock. The two histograms appear significantly different: all the treated cells (a) showed accumulation of fluorescein, while the dead cells (b) did not. The occurrence of fluorochromasia indicated that treatment of HeLa cells with ADR or AD-32 did not induce, in our experimental conditions, any significant loss of cell viability, at the moment when the efflux measurements were performed. Figure 2 shows examples of the time course of fluorescein efflux from control and cells treated with ADR and AD-32, after loading with 2.4 $\mu$M FDA. In all the experiments, the efflux followed first-order kinetics; therefore, the rate constant of the process was considered to be a suitable parameter for evaluating the extent of anthracycline influence on membrane permeability to fluorescein. The decrease in the efflux rate induced by the drugs, was not significantly different when the cells were loaded with fluorescein by using different FDA concentrations. Cells treated with 6 $\mu$M ADR, for instance, showed a rate constant of $1.82 \times 10^{-4}$ ($\pm 0.52$) $s^{-1}$, when incubated with 2.4 $\mu$M FDA, *versus* a constant of $1.65 \times 10^{-4}$ ($\pm 0.56$) $s^{-1}$, when incubated in 4.8 $\mu$M FDA.

**Figure 1** Fluorescence intensity histograms of HeLa cells incubated for 5 min in 2.4 $\mu$M FDA, after drug treatment (a) or thermal shock (b).

When glucose was added to the medium, an increase in the efflux rate was observed, both in control and in treated cells, as shown by the rate constants given in Table I. Taking as the 100% value the rate constant of control cells incubated without glucose, the increase measured was 45% in control cells, 44% and 33% in ADR and AD-32-treated cells, respectively.

**Figure 2** Time course of fluorescein efflux in treated and untreated HeLa cells. Control (○), ADR 2.5 μM (●), 17.2 μM (◆); AD−32 1.3 μM (▲). The fluorescence intensity of each sample was normalized to the value measured at zero time.

**Table I** Influence of glucose on fluorescein efflux rate (23°C) in control and drug-treated HeLa cells.

| *Sample* | *n* | *Rate constant* $(s^{-1}) \times 10^{-4}$ *mean value ± s.d.* | % |
|---|---|---|---|
| Control | 10 | 3.50±0.51 | 100 |
| Control + glucose 20 mM | 3 | 5.06±0.20 | 145[a] |
| ADR 17.2 μM | 3 | 0.66±0.23 | 19 |
| ADR 17.2 μM + glucose 20 mM | 3 | 2.21±0.53 | 63[a] |
| AD-32 1.3 μM | 3 | 1.43±0.49 | 41 |
| AD-32 1.3 μM + glucose 20 mM | 3 | 2.58±0.29 | 74[a] |

[a]Student's *t* test: $P<0.01$

**Figure 3** Dependence of fluorescein efflux rate on drug uptake. The intracellular amount of drug was evaluated in HeLa cells treated for 15 min with: (a) ADR (●), ADR + 20 mM glucose (○); (b) DNR (■), DNR + 20 mM glucose (□); (c) AD−32 (▲), AD-32 + 20 mM glucose (△). Data points are from at least 3 different experiments. For control cells incubated with or without glucose the mean values of 3 and 10 experiments respectively, are reported. Fitted lines were calculated by regression analysis.

Figure 3 shows the dependence of the efflux rate on the drug uptake in cells treated with or without glucose. Both conditions were evaluated, since the addition of glucose in the incubation medium might have induced a further export of the drugs from the cells. For each drug, at a given intracellular concentration, the presence of glucose induced an increase in the rate constant, without any appreciable difference in the trend of the curve. In the range of concentration tested, the efflux rate constant decreased linearly as the amount of intracellular drug increased in ADR-treated cells (a). In DNR-treated cells (b), two different slopes were found: the faster decrease was observed up to an intracellular concentration of ~0.1 nmol mg$^{-1}$ protein. For AD-32 (c), an exponential relationship seems to fit. The greatest inhibition of the efflux was exerted by DNR: the amount that induced a 50% decrease in the efflux rate (0.59 nmol mg$^{-1}$ protein) was lower than that found for ADR and for AD-32 (0.67 and 2.77 nmol mg$^{-1}$ protein, respectively).

Determinations of ATP levels in cells treated with anthracyclines showed that the considered drugs reduced ATP content with respect to control cells and that glucose increased all the values of both treated and control cells. Table II reports ATP levels in cells treated with ADR, DNR and AD-32 at the concentrations that inhibit fluorescein efflux by 50% in respect to the control, both in the presence and in the absence of glucose 20 mM.

The extent of cytotoxicity induced by ADR, AD-32 and DNR in HeLa cells was determined at the same drug concentrations that inhibited the fluorescein efflux. Figure 4 shows the surviving fractions of cells treated for 15 min with ADR (a), DNR (b) and AD-32 (c), as a function of drug availability in the medium. The insert in each panel also shows the amounts of drug taken up by the cells in the same experiments. These data indicate that ADR showed the highest activity: the 50% inhibition of cell proliferation was found at 0.37 nmol mg$^{-1}$ protein for ADR, 0.64 for DNR and 1.40 for AD-32.

### Discussion

The efflux of fluorescein from living cells has been shown to be dependent on membrane integrity and

**Figure 4** HeLa cell survival at 72 h after treatment with ADR (a), DNR (b), and AD-32 (c), plotted *versus* drug concentration in the medium. In the insert of each panel it is shown the amount of drug taken up as a function of concentration in the medium.

**Table II** ATP levels in HeLa cells treated with anthracyclines in the presence or in the absence of glucose.

| | *Without glucose* | | *With glucose* | |
|---|---|---|---|---|
| | *nmol ATP mg$^{-1}$ protein mean value ± s.d. (n = 5)* | % | *nmol ATP mg$^{-1}$ protein mean value ± s.d. (n = 5)* | % |
| Control | 7.64 ± 0.02 | 100 | 13.24 ± 0.60 | 100 |
| ADR 6 μM | 5.50 ± 0.88 | 72 | 10.40 ± 0.22 | 79 |
| DNR 1 μM | 4.78 ± 0.37 | 62 | 11.86 ± 1.25 | 89 |
| AD-32 4 μM | 4.65 ± 0.75 | 61 | 8.16 ± 1.97 | 61 |

on the metabolic energy supply; indeed, the dye release is affected both by membrane-active agents and mitochondrial poisons (Sengbush *et al.*, 1976; Baisch, 1978; Prosperi *et al.*, 1986). Therefore, the monitoring of the kinetics of fluorescence decrease from anthracycline-treated cells may provide information on alterations induced by the drugs, either on membrane structures or energetic cell metabolism. These two aspects can be distinguished, since only the effect on metabolism is offset by glucose.

The anthracyclines considered inhibited fluorescein efflux apparently through the same mechanism shown by metabolic inhibitors, since glucose was able to restore the efflux. This restoration might have been induced by a lower drug accumulation, since anthracyclines are also actively exported from the cells (Danø, 1973; Inaba *et al.*, 1979). However, this possibility is ruled out by the finding that, at the same intracellular amount of drug, fluorescein efflux measured in the presence of glucose was always faster than that in cell incubated without glucose (Figure 3). This increase was also observed in cells incubated without drug, thus suggesting that glucose increases energy levels available for efflux. This hypothesis is supported by the finding that drug treatments also induced a reduction of the ATP levels into the cells. Concurrently, glucose was able to increase energy availability, both in treated and in control cells. Furthermore, the possibility of an inhibition of fluorescein efflux by a direct interference of anthracycline molecules, seems rather unlikely since the rate constant of the dye release did not change significantly when higher levels of fluorescein were attained into the cells by using different FDA concentrations. It appears therefore that ADR, DNR and AD-32, irrespective of their ability to bind to DNA, affected membrane permeability functions dependent on the metabolic energy supply, by impairing mitochondrial activity. Interactions at the mitochondrial level, resulting in impaired metabolic reactions have been already observed by several authors (Gosalvez *et al.*, 1974; Iwamoto *et al.*, 1974; Ferrero *et al.*, 1976; Mailer & Petering, 1976; Folkers *et al.*, 1977; Goormaghtigh *et al.*, 1982; Solaini *et al.*, 1985) and our findings are consistent with these reports.

The anthracyclines investigated in this study showed a different ability in inhibiting fluorescein efflux. In particular, AD-32 was at least 4 times less effective than DNR and ADR, when the rate constant was related to the amount of drug accumulated intracellularly. These results could be explained by taking into consideration a possible differential affinity of the drugs for mitochondrial structures. Indeed, binding to cellular compartments is greatly established by the charged form of the anthracyclines, that are protonated in the amino group of the sugar moiety (Skovsgaard & Nissen, 1982; Siegfried *et al.*, 1985). AD-32 is less ionizable than DNR and ADR, due to the substitution of an hydrogen with a -$COCF_3$ group in the amino residue; therefore, it is possible that mitochondrial uptake, that could be also driven, like other cationic dyes (Johnson *et al.*, 1981; Lampidis *et al.*, 1983), by the inside-negative potential of mitochondrial membrane, is lower for the uncharged form of the drugs. Furthermore, it is known that ionized anthracyclines interact with negatively charged phospholipids (i.e. cardiolipin, Goormaghtigh *et al.*, 1980) thereby impairing the cytochrome c oxidase activity (Goormaghtigh *et al.*, 1982). These arguments may account both for the qualitative and quantitative differences found for the relationship between fluorescein efflux rate and intracellular amount of drug.

Although many reports support an interaction of anthracyclines with energetic metabolism, the actual contribution of these interactions to cytotoxicity is not yet known, nor the relationship between cytotoxicity and changes in membrance functions has been studied extensively. A correspondence of ADR doses required both for cytotoxicity and changes in the rate of lectin-induced cellular agglutination has been reported by Murphree *et al.* (1976); on the other hand, inhibition of $^{86}Rb$ uptake in confluent fibroblasts has been shown to occur at concentrations two orders of magnitude higher than those causing cytotoxicity (Landolph *et al.*, 1980). In our experiments, the same conditions that affected permeability properties were also toxic to HeLa cells. The cytotoxicity assay showed that for each drug, the amount reducing cell survival to 50% was in the same order of magnitude as that inhibiting fluorescein efflux to the same degree. It is worth noting that the effect on membrane permeability was assessed immediately, while the cells were still viable. Cytotoxicity was, on the contrary, evaluated 72 h later, when interaction with other cellular targets, the metabolic fate of the drugs, as well as, possibly, recovery of the cells had potentially occurred. These findings suggest a further study on fluorescein efflux evaluated at different times after drug treatment, in relation to other cellular parameters as ATP content and cell cycle distribution, in order to better clarify to what extent modification of these factors contributes to anthracycline cytotoxicity.

We thank Mr. G. Michelazzo for drawings and photographic work, and Miss A. Ganassa for typing the manuscript. Work supported by C.N.R., Special Project 'Oncology'.

### References

AUGSTEN, K. & STRAUSS, D.G. (1984). The effects of violamycins on the cell membranes of murine macrophages and L 1210 tumour cells *in vivo*. *Studia Biophysica*, **104**, 219.

BAISCH, H. (1978). Effects of X-rays on cell membranes. II. Changes of permeability measured by fluorescein efflux. *Rad. Environ. Biophys.*, **15**, 221.

CHAHWALA, S.B. & HICKMAN, J.A. (1985). Investigations of the action of the antitumor drug adriamycin on tumour cell membrane function-I. *Biochem. Pharmacol.*, **34**, 1501.

DANØ, K. (1973). Active outward transport of daunomycin in resistant Ehrlich ascite tumour cells. *Biochim. Biophys. Acta*, **323**, 466.

DI MARCO, A., ARCAMONE, F. & ZUNINO, F. (1974). Daunomycin (daunorubicin) and adriamycin and structural analogues: biological activity and mechanism of action. In *Antibiotics*, (eds) Corcoran, J. and Hahnm, F.E., Vol. III, p. 101. Springer-Verlag: Berlin, Heidelberg, New York.

FERRERO, M.E., FERRERO, E., GAJA, G. & BERNELLI-ZAZZERA, A. (1976). Adriamycin energy metabolism and mitochondrial oxidations in the heart of treated rabbits. *Biochem. Pharmacol.*, **25**, 125.

FOLKERS, K., LIU, M., WATANABE, T. & PORTER, T.H. (1977). Inhibition by adriamycin of the mitochondrial biosynthesis of coenzyme $Q_{10}$ and implication for the cardiotoxicity of adriamycin in cancer patients. *Biochem. Biophys. Res. Commun.*, **77**, 1536.

GOLDMAN, R., FACCHINETTI, T., BACH, D., RAZ, A. & SHINITZKY, M. (1978). A differential interaction of daunomycin, adriamycin and their derivatives with human erythrocytes and phospholipids bilayers. *Biochim. Biophys. Acta*, **512**, 245.

GOORMAGHTIGH, E., BRASSEUR, R. & RUYSSCHAERT, J.M. (1982). Adriamycin inactivates cytochrome c oxidase by exclusion of the enzyme from its cardiolipin essential environment. *Biochem. Biophys. Res. Commun.*, **104**, 314.

GOORMAGHTIGH, E., CHATELAIN, P., CASPERS, J. & RUYSSCHAERT, J.M. (1980). Evidence of a specific complex between adriamycin and negatively-charged phospholipids. *Biochim. Biophys. Acta*, **597**, 1.

GOORMAGHTIGH, E. & RUYSSCHAERT, J. (1984). Anthracycline glycoside-membrane interactions. *Biochim. Biophys. Acta*, **779**, 271.

GOSALVEZ, M., BLANCO, M., HUNTER, J., MIKO, M. & CHANCE, B. (1974). Effects of anticancer agents on the respiration of the isolated mitochondria and tumor cells. *Eur. J. Cancer*, **10**, 567.

INABA, M., KOBAYASHI, H., SAKURAI, Y. & JOHNSON, K. (1979). Active efflux of daunorubicin and adriamycin in sensitive and resistant sublines of P388 leukemia. *Cancer Res.*, **39**, 1365.

ISRAEL, M., MODEST, E.J. & FREI III, E. (1975). N-trifluoroacetyladriamycin-14-valerate, an analog with greater experimental antitumor activity and less toxicity than adriamycin. *Cancer Res.*, **35**, 1365.

IWAMOTO, Y., HANSEN, I.L., PORTER, T.H. & FOLKERS, K. (1974). Inhibition of coenzyme $Q_{10}$-enzymes, succinoxidase and NADH-oxidase, by adriamycin and other quinones having antitumor activity. *Biochem. Biophys. Res. Commun.*, **58**, 633.

JOHNSON, L.W., WALSH, M.L., BOCKUS, B.J. & CHEN, L.B. (1981). Monitoring of relative mitochondrial membrane potential in living cells by fluorescence microscopy. *J. Cell Biol.*, **88**, 526.

KRISHAN, A., ISRAEL, M., MODEST, E. & FREI III, E. (1976). Differences in cellular uptake and cyto-fluorescence of adriamycin and N-trifluoroacetyl-adriamycin-14-valerate. *Cancer Res.*, **36**, 2108.

LAMPIDIS, T.J., BERNAL, S.D., SUMMERHAYES, I.C. & CHEN, L.B. (1983). Selective toxicity of rhodamine 123 in carcinoma cells *in vitro*. *Cancer Res.*, **43**, 716.

LANDOLPH, J.R., BHATT, R.S., TELFER, N. & HEIDELBERGER, C. (1980). Comparison on adriamycin- and ouabain-induced cytotoxicity and inhibition of $^{86}$rubidium transport in wild-type and ouabain-resistant CEH/10T1/2 mouse fibroblasts. *Cancer Res.*, **40**, 4581.

LOWRY, O.H., ROSEBROUGH, N.J., FARR, A.L. & RANDALL, R.J. (1951). Protein measurement with the Folin phenol reagent. *J. Biol. Chem.*, **193**, 265.

MAILER, K. & PETERING, D.H. (1976). Inhibition of oxidative phosphorylation in tumor cells and mitochondria by daunomycin and adriamycin. *Biochem. Pharmacol.*, **25**, 2085.

MURPHREE, S.A., CUNNINGHAM, L.S., HWANG, K.M. & SARTORELLI, A.C. (1976). Effects of adriamycin on surface properties of sarcoma 180 ascite cells. *Biochem. Pharmacol.*, **25**, 1227.

MURPHREE, S.A., TRITTON, T.R., SMITH, P.L. & SARTORELLI, A.C. (1981). Adriamycin-induced changes in the surface membrane of Sarcoma 180 Ascites cell. *Biochim. Biophys. Acta*, **649**, 317.

PROSPERI, E., CROCE, A.C., BOTTIROLI, G. & SUPINO, R. (1985). Influence of daunorubicin on membrane permeability properties: detection by means of intra-cellular accumulation and efflux of fluorescein. *Chem.-Biol. Interactions*, **54**, 271.

PROSPERI, E., CROCE, A.C., BOTTIROLI, G. & SUPINO, R. (1986). Flow cytometric analysis of membrane permeability properties influencing intracellular accumulation and efflux of fluorescein. *Cytometry*, **7**, 70.

ROGERS, K.L., CARR, B.I. & TÖKES, A. (1983). Cell surface-mediated cytotoxicity of polymer-bound adriamycin against drug-resistant hepatocytes. *Cancer Res.*, **43**, 2741.

ROTMAN, B. & PAPERMASTER, B.W. (1966). Membrane properties of living mammalian cells as studied by enzymatic hydrolysis of fluorogenic esters. *Proc. Natl. Acad. Sci. USA.*, **55**, 134.

SENGBUSH, G.V., COUWENBERGS, C., KÜNHER, J. & MÜLLER, U. (1976). Fluorogenic substrate turnover in single living cells. *Histochem. J.*, **8**, 341.

SIEGRIED, J.M., BURKE, T.M. & TRITTON, T.R. (1985). Cellular transport of anthracyclines by passive diffusion. *Biochem. Pharmacol.*, **34**, 593.

SKOVSGAARD, T. & NISSEN, N.I. (1982). Membrane transport of anthracyclines. *Pharmac. Ther.*, **18**, 293.

SOLAINI, G., RONCA, G. & BERTELLI, A. (1985). Inhibitory effects of several anthracyclines on mitochondrial respiration and coenzyme $Q_{10}$ protection. *Drugs Exptl. Clin. Res.*, **8**, 533.

SONTAG, W. (1977). A comparative kinetic study on the conversion of fluoresceindiacetate to fluorescein in living cells and *in vitro*. *Rad. Environ. Biophys.*, **14**, 1.

STEEN, H.B. & LINDMO, T. (1979). Flow cytometry: a high resolution instrument for everyone. *Science*, **204**, 403.

TRITTON, T.R., YEE, G. & WINGARD, JR. L.B. (1983). Immobilized adriamycin a tool for separating cell surface from intracellular mechanism. *Fed. Proc.*, **42**, 284.

WIENER, S., WIENER, R., URIVETZKY, M. & MEILMAN, E. (1974). Coprecipitation of ATP with potassium perchlorate: the effect on the firefly enzyme assay of ATP in tissue and blood. *Anal. Biochem.*, **59**, 489.